# Primary Benign Neoplasms of the Spine

**DOI:** 10.3390/diagnostics13122006

**Published:** 2023-06-08

**Authors:** Sisith Ariyaratne, Nathan Jenko, Karthikeyan P. Iyengar, Steven James, Jwalant Mehta, Rajesh Botchu

**Affiliations:** 1 Department of Musculoskeletal Radiology, Royal Orthopedic Hospital, Birmingham B31 2AP, UK; sisith.ariyaratne@nhs.net (S.A.); nathan.jenko@nhs.net (N.J.); stevenjames@nhs.net (S.J.); 2 Department of Orthopedics, Southport and Ormskirk Hospital NHS Trust, Southport PR8 6PN, UK; kartikp31@hotmail.com; 3 Department of Spinal Surgery, Royal Orthopedic Hospital, Birmingham B31 2AP, UK; jwalantmehta@nhs.net

**Keywords:** benign primary vertebral tumours, osteoma, osteoid osteoma, osteoblastoma, fibrous dysplasia, osteochondroma, chondroblastoma, haemangioma, simple bone cyst, aneurysmal bone cyst, giant cell tumour, notochordal rest, eosinophilic granuloma, MRI, CT

## Abstract

Benign tumours comprise the majority of primary vertebral tumours, and these are often found incidentally on imaging. Nonetheless, accurate diagnosis of these benign lesions is crucial, in order to avoid misdiagnosis as more ominous malignant lesions or infection. Furthermore, some of these tumours, despite their benign nature, can have localised effects on the spine including neural compromise, or can be locally aggressive, thus necessitating active management. Haemangiomas and osteomas (enostosis) are the commonest benign tumours encountered. Others include osteoid osteoma, osteoblastoma, fibrous dysplasia, osteochondroma, chondroblastoma, haemangioma, simple bone cysts, aneurysmal bone cysts, giant cell tumours, eosinophilic granuloma and notochordal rests. The majority of lesions are asymptomatic; however, locally aggressive lesions (such as aneurysmal bone cysts or giant cell tumours) can present with nonspecific symptoms, such as back pain, neurological deficits and spinal instability, which may be indistinguishable from more commonly encountered mechanical back pain or malignant lesions including metastases. Hence, imaging, including radiography, computed tomography (CT) and magnetic resonance imaging (MRI), plays a critical role in diagnosis. Generally, most incidental or asymptomatic regions are conservatively managed or may not require any follow-up, while symptomatic or locally aggressive lesions warrant active interventions, which include surgical resection or percutaneous treatment techniques. Due to advances in interventional radiology techniques in recent years, percutaneous minimally invasive techniques such as radiofrequency ablation, sclerotherapy and cryoablation have played an increasing role in the management of these tumours with favourable outcomes. The different types of primary benign vertebral tumours will be discussed in this article with an emphasis on pertinent imaging features.

## 1. Introduction

Primary vertebral tumours are rare, and benign tumours constitute the majority of these. It is estimated that the incidence of haemangiomas and osteomas, which were found to be the most common primary tumours of the spine, is between 11% and 14% [1]. These commonly seen benign lesions are often asymptomatic, diagnosed incidentally and do not require any active intervention. Certain other types of lesions, albeit benign, can be locally aggressive and, as such, warrant active management. The diagnosis of the latter can be challenging, both due to their rarity as well as the nonspecific nature of presentation, most commonly back pain [2].

Early detection and accurate diagnosis are important for effective management, particularly those lesions that have the potential to be locally aggressive. Imaging, particularly Computed Tomography (CT) and Magnetic Resonance Imaging (MRI), plays a crucial role in diagnosis of these conditions, and having a sound understanding of the imaging principles and characteristic features of different types of tumours is paramount both for correct diagnosis and preventing misdiagnosis as malignant tumours or infection. In the case of benign lesions, accurate and confident diagnosis on imaging can prevent the need for unnecessary biopsy and intervention.

Treatment options depend on the extent and type of tumour, but in a broad sense, the vast majority of lesions are conservatively managed and surgical resection is reserved for highly locally aggressive lesions which may compromise adjacent neural structures. Percutaneous minimally invasive interventions which are typically performed under image guidance may play a role in the management of the latter types of lesions in some instances, and include radiofrequency ablation, sclerotherapy and cryoablation. When active intervention is required, this is further complicated due to the complex anatomy of the spine and risk of damage to critical structures which are in close proximity, particularly the spinal cord [3,4].

This review article aims to discuss the different types of primary benign tumours of the vertebral column, with emphasis on the approach to imaging and distinguishing imaging features. Discussion of secondary tumours, rare malignant varieties of some of these tumours and lesions of infective aetiology will be excluded.

## 2. Imaging Modalities and Techniques

The imaging modalities and techniques have been previously discussed in an article pertaining to primary malignant vertebral tumours, and the broad principles discussed are identical to those utilized in the imaging of benign vertebral lesions.

## 3. Types of Lesions

The various types of primary benign tumours of the spine can be classified based on the World Health Organisation (WHO) classification of bone tumours (Table 1).

### 3.1. Osteoma (Enostosis)

Osteoma, also known as a bone island or ivory osteoma, is one of the commonest primary benign vertebral tumours, detected in approximately 14% of cadavers according to one study [2] and present in 1.4% of the population according to another source [3]. While they can be anywhere in the skeleton, osteomas have a propensity for the axial skeleton. In the spine, they most frequently occur in the thoracic and lumbar spine and involve the vertebral body [2]. They are developmental lesions (not present at birth), and histologically comprise dense cortical bone within the medullary cavity, usually adjacent to the cortex or an endplate [2,3]. They are often identified incidentally and are asymptomatic.

**Figure 1:**
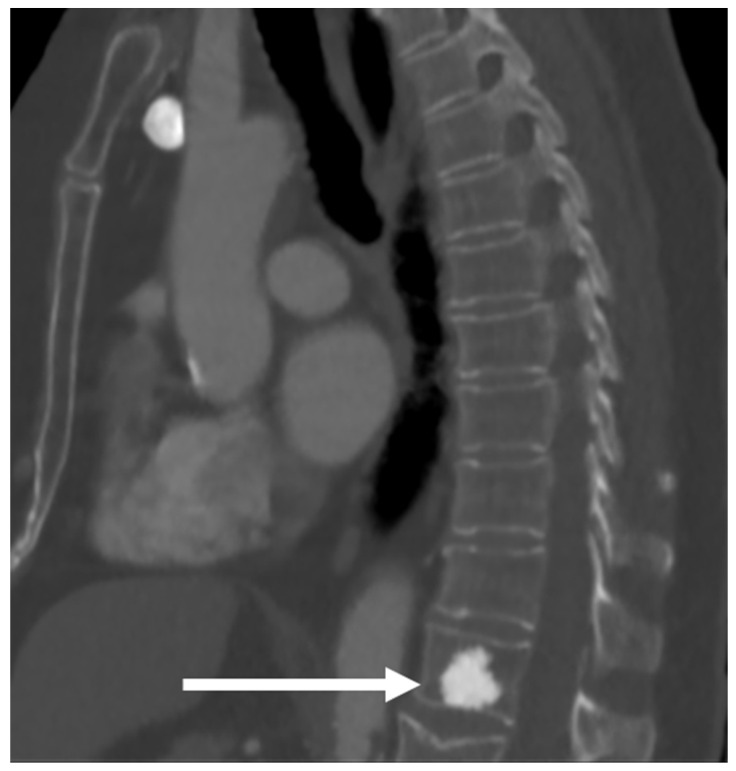
Sagittal CT demonstrating an osteoma of T12 vertebra (white arrow). Note the typical sclerotic, hyperdense appearance with spiculated margins.

These lesions present as densely sclerotic and well-demarcated lesions on imaging, often 2–10 mm in size. They can have spiculated margins. Occasionally, they can be larger, measuring 2–3 cm (termed giant bone islands), and some can also grow with age. Multiple lesions are seen in about a fifth of cases, and while the vast majority are sporadic, they can also occur in the setting of osteopoikilosis, an inherited sclerosing bone dysplasia characterised by multiple osteomas; however, in osteopoikilosis, the lesions are periarticular [5]. On radiographs and CT scans, these lesions appear markedly hyperdense compared to the adjacent bone [3] (Figure 1). Measurement of the CT Hounsfield units (HU) of these lesions can be helpful in distinguishing them from osteoblastic metastases which may have a similar appearance. As a general rule, osteomas are denser, with mean and maximum CT attenuation values of 1190 ± 239 HU and 1323 ± 234 HU, respectively, and those of osteoblastic metastases are 654 ± 176 HU and 787 ± 194 HU, respectively [6]. A cut-off of 885 HU for mean attenuation has a 95% sensitivity and 96% specificity for osteomas, and a cut-off of 1060 HU for maximum CT attenuation has a 95% sensitivity and 96% specificity according to one study, although it is by no means definitive [6].

**Figure 2:**
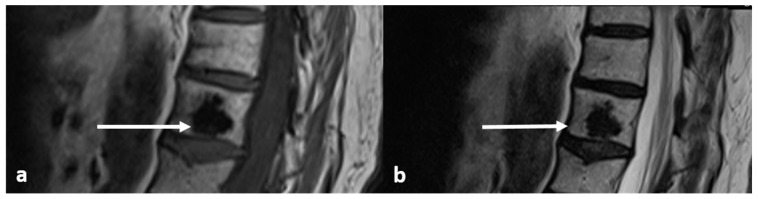
Sagittal T1 (**a**) and T2 (**b**) images demonstrating the same osteoma as Figure 1 (white arrows). The low T1 and T2 signal is a typical feature.

On MRI scans, the lesions are devoid of signal on T1 and T2 sequences, appearing hypointense, lacking any surrounding marrow oedema and not demonstrating increased diffusion restriction or contrast enhancement (Figure 2a,b).

They do not usually require any intervention or follow-up. While the majority remain stable in size over time, approximately a third can increase in size.

### 3.2. Osteoid Osteoma

Osteoid osteomas (OOs) account for 10–14% of primary vertebral tumours. The majority are seen the lumbar spine, followed by the cervical spine, thoracic spine and sacrum. They have a particular predilection for the posterior elements, with 75–90% of OOs occurring here [3,7]. It commonly affects a younger population, most patients being under 30 years at presentation [3,7]. The classic presentation is back pain, particularly nocturnal pain. It can also cause scoliosis, and when it does, the OO is usually located at the concavity [8]. There may be radicular symptoms due to a local inflammation reaction from the lesion irritating adjacent nerve roots [3].

**Figure 3:**
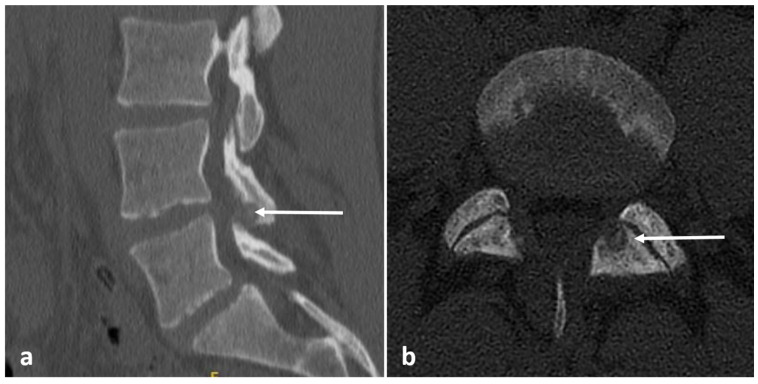
Sagittal (**a**) and axial (**b**) CT images showing an osteoid osteoma of the left L4 lamina (white arrows). The lucent nidus with central sclerotic ‘dot’ is well demonstrated. The location in the neural arch is also typical for OO.

**Figure 4:**
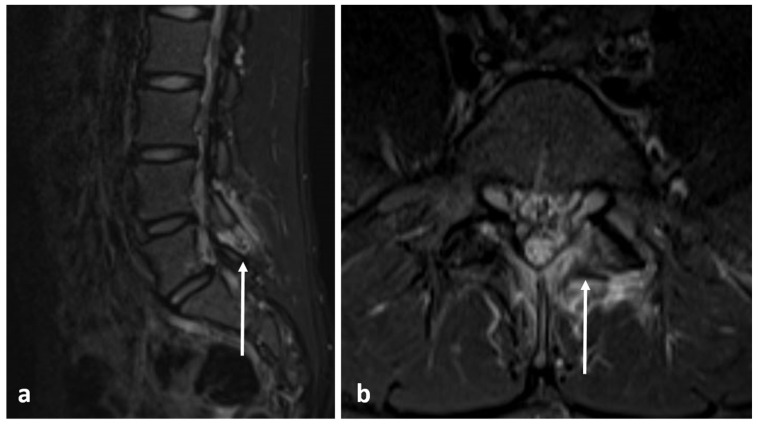
Sagittal (**a**) and axial (**b**) STIR images demonstrating the same osteoid osteoma showed in Figure 3a,b (white arrows). High STIR signal corresponding to oedema is typical. The nidus is not clearly seen in MRI.

Radiographs have a limited role in diagnosis. The presence of a lucent nidus surrounded by sclerotic reactive bone is usually pathognomonic for OOs. A central sclerotic dot may also be present. The nidus is typically <1.5 to 2 cm in diameter. These features are best demonstrated via CT (Figure 3a,b). It is worth noting, however, that reactive bone sclerosis surrounding the nidus is less of a feature in spinal OOs compared to those occurring in the appendicular skeleton. On rare occasions, multiple niduses can be present. MRI, while extremely sensitive, may be unable to identify the nidus due to the presence of surrounding marrow oedema (Figure 4a,b), which can present a pitfall, as using MRI alone may result in the lesion being misdiagnosed as an aggressive bone lesion or a stress fracture. As such, a combination of both CT and MRI is useful. The nidus is typically of low to intermediate T1 signal, variable T2 signal and may contain areas of pseudo-signal void due to mineralisation. The nidus may also show variable enhancement in post contrast imaging [3,7].

**Figure 5:**
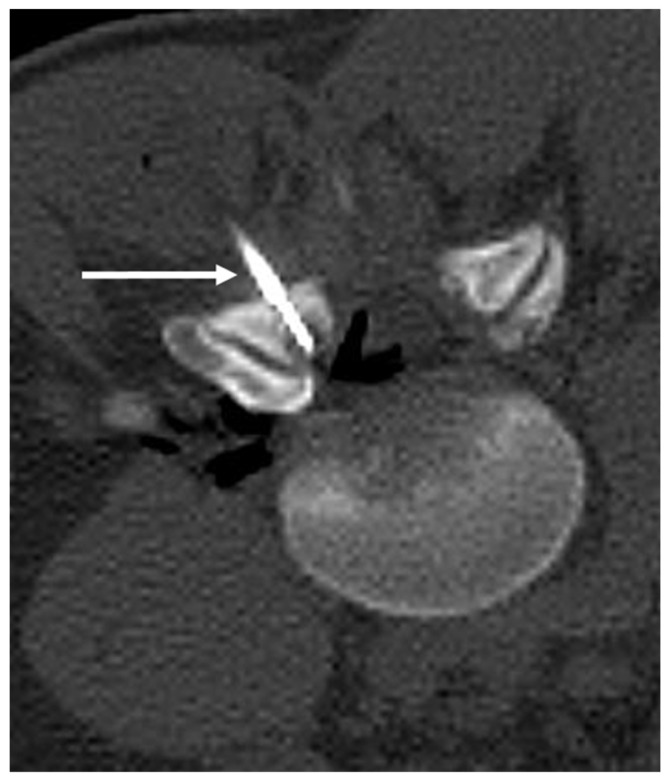
Axial CT image obtained during ablation of the osteoid shown in Figure 3 and Figure 4. Note the electrode placement (white arrow) with tip within the nidus. Air has been injected into the epidural space for neuroprotection (black).

OOs can spontaneously resolve with medical management. Surgery has been used for curative management; however, percutaneous radiofrequency ablation has been used in recent times and has been shown to be an effective and safe alternative, with low complication and recurrence rates and a reduction in hospitalisation [9,10]. The procedure is best performed under CT guidance. Injection of air into the epidural space may be beneficial during the procedure for neuroprotection to prevent damage to thecal sac [10] (Figure 5).

### 3.3. Osteoblastoma

Osteoblastoma is a rare osteogenic tumour which is histologically similar to OO, the main differences being that osteoblastomas are larger, measuring >1.5–2 cm, and unlike OOs, they do not undergo spontaneous resolution and have the potential to be locally aggressive and can extend beyond the cortex [3]. The demographics are similar to those of OOs. They also have an affinity for the posterior elements [11], and commonly involve the lumbar spine, followed by thoracic and cervical spines, with the sacrum being least commonly involved [3]. Extension from the posterior elements into the vertebral body can be seen in about a third of the cases [11]. Pain is the commonest symptom, and painful scoliosis is a recognized presentation. Neurological symptoms can occur when there is extraosseous extension impinging on neural structures [11].

**Figure 6:**
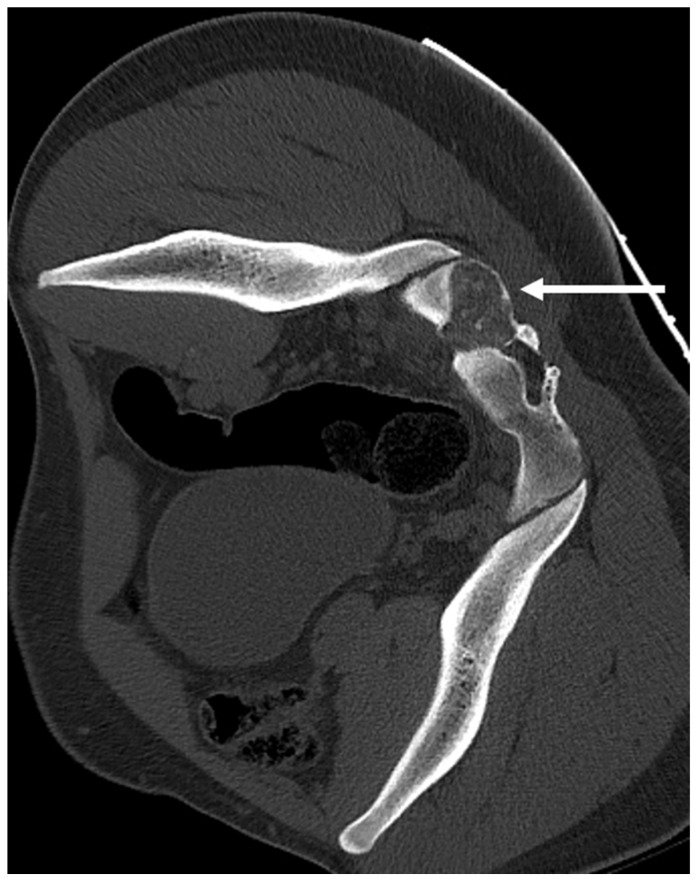
Axial CT image of an osteoblastoma of the sacrum (white arrow). Note the typical lytic and slightly expansile appearance and intralesional calcification.

**Figure 7:**
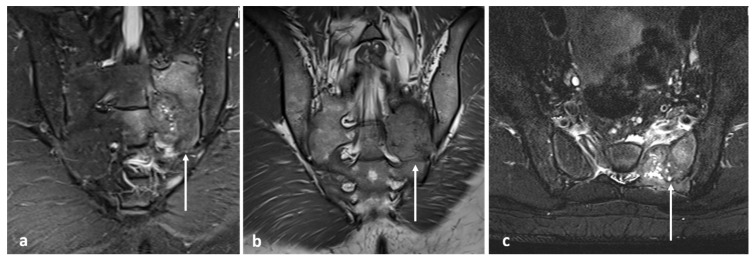
Coronal STIR (**a**), coronal T2 (**b**) and axial STIR (**c**) images showing the osteoblastoma of sacrum (white arrows). The lesion shows intermediate to high T2 signal.

Imaging features can be non-specific. The lesions are typically lucent on radiographs and CT (Figure 6). There can be variable ossification of the matrix, which can manifest as intralesional calcification. The lesions can be expansile and may have a sclerotic rim. Cortical destruction and extraosseous extension into the paravertebral region and spinal canal are seen in more aggressive cases [11,12]. In MRI, osteoblastoma usually has a low to intermediate T1 signal and an intermediate to high T2 signal (Figure 7a–c), and may show a variable degree of enhancement with contrast. Due to associated significant inflammatory response, it is also fairly common to see extensive surrounding soft tissue oedema on fluid-sensitive sequences. Bone scintigraphy is also very sensitive, as these tumours show avid uptake of Technetium-99 [11].

The mainstay of management involves surgical resection and curettage, and in the case of high aggressive lesions, en-bloc resection may be performed, as these tumours have a high rate of recurrence. In a proportion of selected cases, percutaneous image-guided radiofrequency or cryoablation may be utilised [13]. As with OO ablation, neuroprotection through injecting epidural air to prevent damage to the thecal sac can be beneficial [10].

### 3.4. Fibrous Dysplasia

Fibrous dysplasia (FD) is a fibro-osseous lesion of the bone characterised by metaplastic replacement of the medullary component with fibrous tissue and irregular osteoid formation, and commonly presents in the second to third decades [14]. It can be monostotic or polyostotic and can be seen in association with several syndromes. FD is extremely rare in the spine, with only a few cases reported in the literature, and when it does occur, it is more commonly associated with polyostotic disease with concurrent involvement of the appendicular skeleton [3,14]. The thoracolumbar spine is most commonly affected [14]. Presentation ranges from asymptomatic to symptomatic, with symptoms including backpain, spinal deformity and neurological symptoms.

**Figure 8:**
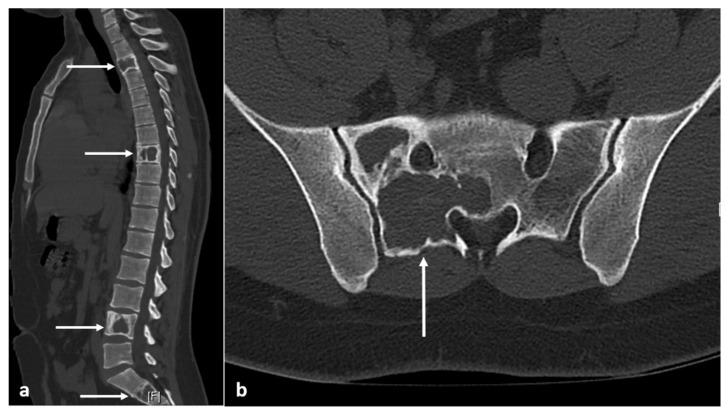
Sagittal (**a**) and axial (**b**) CT images demonstrating fibrous dysplasia of thoracic and lumbar vertebrae and sacrum (white arrows). The ground glass matrix is not always seen, and some such as this can appear lytic with thin peripheral cortical shell.

**Figure 9:**
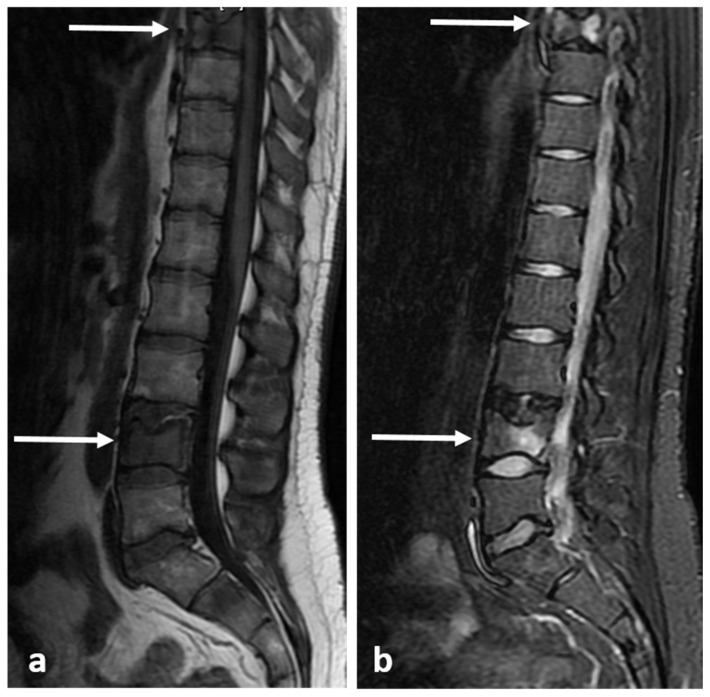
Sagittal T1 (**a**) and STIR (**b**) images showing fibrous dysplasia of thoracic and lumbar vertebrae (white arrows). The lesions have intermediate to low T1 and intermediate to high T2 signal.

FD can have a variable degree of intralesional ossification and cystic degeneration, which determines the imaging characteristics. The characteristic appearance is the presence of intralesional ossification with a ‘ground-glass’ matrix, which is best demonstrated in CT [3,15]. It can also present as a lytic expansile lesion with a preserved thin cortical shell (Figure 8a,b). MRI characteristics of fibrous dysplasia are variable, typically showing signal intensity that is intermediate to low on T1-weighted images and intermediate to high on T2-weighted images, the degree of T2/fluid signal being contingent on the degree of ossification and cystic degeneration (Figure 9a,b), with higher fluid signal seen in the presence of cystic change. Involvement of an adjacent vertebra or rib is also a useful diagnostic clue. CT-guided biopsy may be required for confirmation in cases where imaging is equivocal.

Treatment depends on the presence of symptoms. Surgery may be required to correct deformities, prevent pathologic fracture, and resect symptomatic lesions. Very rarely, malignant transformation, usually into osteosarcoma, can occur [14].

### 3.5. Osteochondroma

Osteochondromas are the commonest benign bone lesion [16], most commonly occurring in the long bones. Multiple osteochondromas are usually associated with Hereditary Multiple Exostosis (HME). Only 3–4% of osteochondromas occur in the spine, although this may rise to as high as 9% with HME [17,18]. The majority of spinal osteochondromas, approximately 50%, occur in the cervical spine [18].

**Figure 10:**
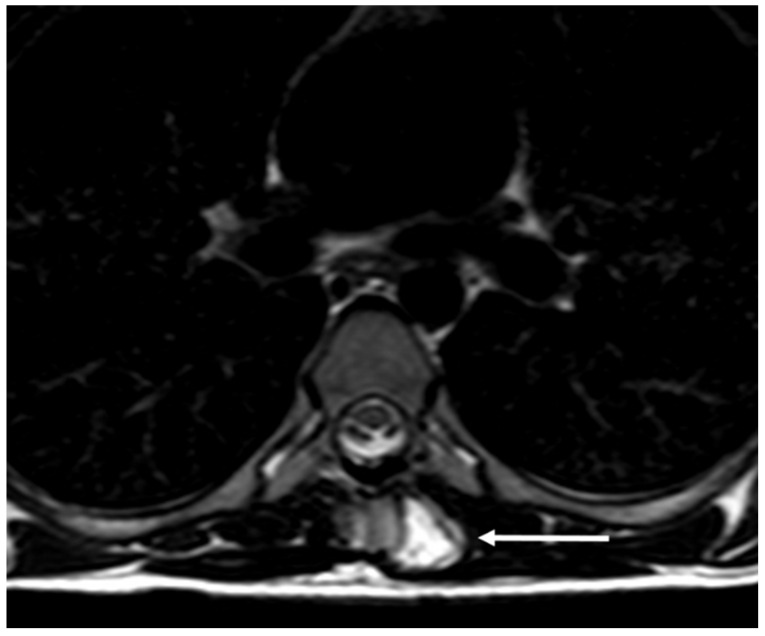
Axial T2 image showing a thoracic vertebral osteochondroma with high T2 signal cartilage cap (white arrow).

**Figure 11:**
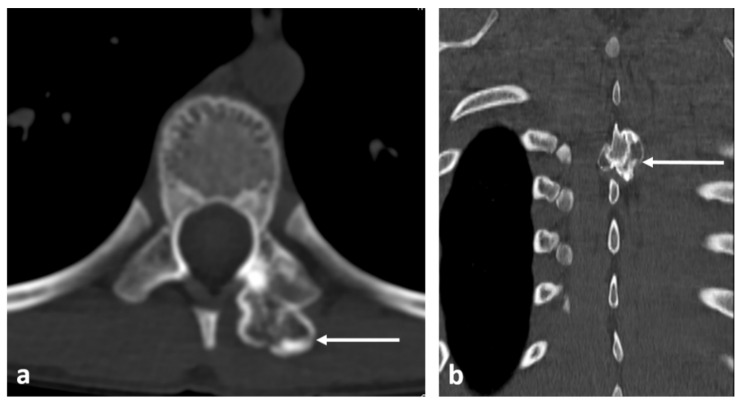
Axial (**a**) and coronal (**b**) CT images demonstrating a pedunculated osteochondroma of a thoracic vertebra (white arrows). Well defined lesion with continuity with cortex and medulla are typical features.

The morphology of osteochondroma can be best visualized using MR. The osseous component is continuous with the bone marrow and will exhibit the same signal characteristics as the adjacent bone marrow. The cartilaginous cap usually returns high T2 signal as it is comprised of hyaline cartilage, although this may be variable if the cap becomes calcified (Figure 10). Cartilage is frequently lined with a thin low-signal line representing the intact perichondrium [19]. Osteochondromas may also be identified in CT due to the characteristic sessile or pedunculated osseous component (Figure 11a,b). The cartilage cap will only be visible in CT when calcified. Appearance on bone scintigraphy is variable; the presence of uptake cannot differentiate between endochondral ossification or malignant transformation [20].

Osteochondromas are usually asymptomatic. Symptoms frequently arise due to mass effect on adjacent structures. Hence, spinal osteochondromas can be particularly troublesome. Numerous case reports have been published due to compression from osteochondromas; complications remain very rare, but multiple case reports of radiculopathy [16], central cord compression [21], vertebral artery compression/occlusion [22] and dysphagia [23,24] have been published. In a study by Jackson et al., out of 227 patients with HME, only 8 patients were found to have spinal osteochondromas, only 1 of which was symptomatic. Notably, pelvic and rib osteochondromas were associated with spinal lesions [25].

**Figure 12:**
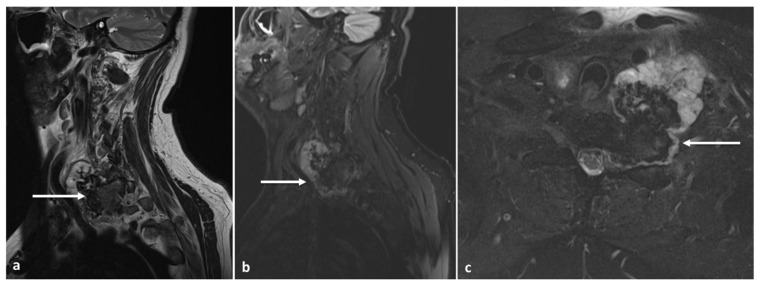
Sagittal T2 (**a**), STIR (**b**) and axial STIR (**c**) images showing a cervical vertebral osteochondroma with transforamtion into chondrosarcoma (white arrows).

The cartilaginous cap can degrade into a secondary chondrosarcoma with reported frequencies of approximately 1%; in HME, this may rise to as high as 9% due to the higher number of lesions [20] (Figure 12a–c). Hence, new pain in the context of HME should be promptly investigated with MR. Multiple cut-offs for cartilage cap thickness have been proposed, ranging between 1.5 cm and 3 cm. Bernard et al. found that a cut-off of 2 cm was 100% sensitive and 98% specific for MR in their cohort [26]. Additionally, in MR, chondrosarcoma exhibits septal enhancement in addition to peripheral enhancement [27] as well as earlier enhancement [28]. To the best of our knowledge, contrast-enhanced assessment has not been widely adopted.

### 3.6. Chondroblastoma

Vertebral chondroblastomas are exceedingly rare. The largest case series from the Mayo clinic notes that only 9 out of 856 chondroblastomas at their institution occurred in the spine [29]. By 2017, only 30 cases have been published in the literature [30].

Chondroblastomas classically affect the epiphysis of an immature skeleton. When occurring in the vertebra, the lesions often arise later in life—the mean reported age at diagnosis is 31.9 years [31]. Radiologically, chondroblastomas are inseparable from other lesions. Chondroblastomas are osteolytic, but otherwise, appearances are highly variable. Reported cases include aggressively appearing lesions with a soft-tissue mass [31] as well as non-expansile lytic lesions [32]. The presence of calcification is variable with reported cases ranging from pronounced to subtle [29], which can appear similar to chondrosarcoma. Secondary aneurysmal bone cyst formation and adjacent bone marrow oedema may also be present. Lesions are vascular and enhance after contrast administration [30]. Treatment is surgical, but recurrence is frequent, with reports ranging from 10% [30] to greater than 40% [29]. Percutaneous radiofrequency ablation can also be performed in some instances.

### 3.7. Haemangioma

Haemangioma constitutes the most common primary benign tumour of the spine, identified in 11% of spines post mortem [33]. They commonly involve the vertebral body, and involvement of the posterior elements is rare [34]. Approximately half of them involve the thoracic spine, while the latter occur in the cervical and lumbar spine. Sacral involvement is rare. They are hamartomatous lesions comprising thin-walled vessels and sinuses lined by endothelium interspersed within the trabecular bone and contain fat [34]. The lesions are often asymptomatic and identified incidentally. Only a minority of them, approximately 1%, are symptomatic, with the commonest presentation being back pain, and sometimes may be attributed to pathological fracture in the case of large haemangiomas [3,33]. Those that have paraspinal or posterior element involvement, or a lack a large fatty stroma are most likely to be symptomatic. Rarely, haemangiomas can be expansile, resulting in enlargement of the affected vertebra and causing compression of neural structures, an entity known as aggressive haemangioma [3]. These are most commonly seen in the thoracic spine.

**Figure 13:**
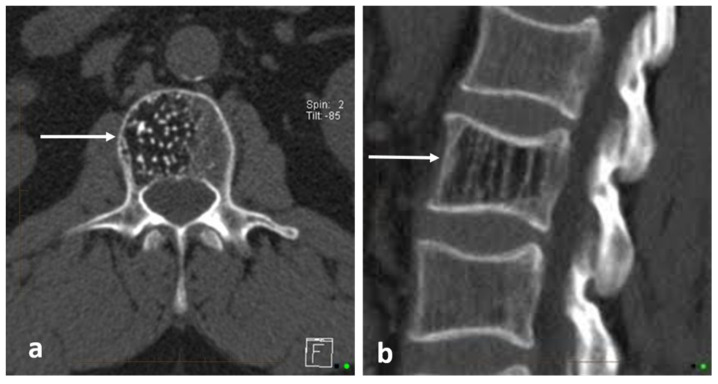
Axial (**a**) and sagittal (**b**) CT images demonstrating a lumbar vertebral haemangioma (white arrows). Note typical ‘polka dot’ appearance on axial image, and the ‘corduroy’ pattern on sagittal image.

Conventional haemangiomas have a classical appearance on imaging, which enables confident diagnosis. In CT, they appear as lesions confined to the vertebral body, and have a vertically oriented thickened trabecualae (termed the ‘corduroy sign’ due to its resemblance of a corduroy fabric) with a honeycomb appearance, best appreciated on sagittal imaging, and cause rare fraction of the bone (Figure 13b). In axial CT, this appears as foci of trabecular bone interwoven between the fatty marrow and vascular lacunae, giving rise to a ‘polka dot’ pattern [3] (Figure 13a).

**Figure 14:**
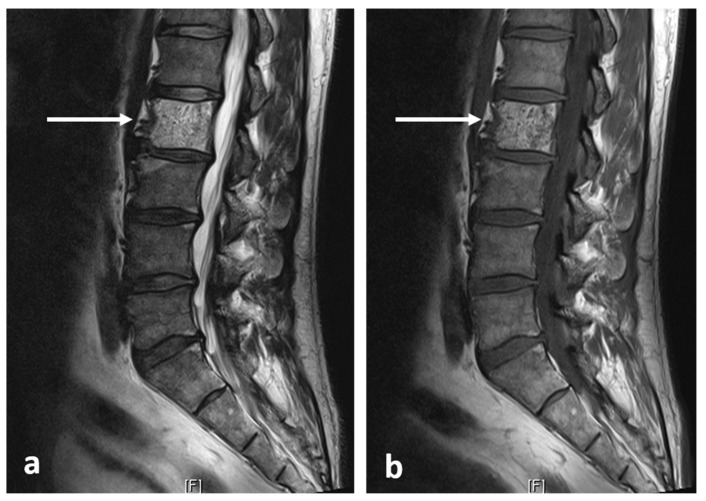
Sagittal T2 (**a**) and T1 (**b**) images demonstrating a typical haemangioma in L2 vertebra (white arrows). The high T1 and T2 signal is characteristically seen in typical haemangiomas due to presence of fat and slow vascular flow.

**Figure 15:**
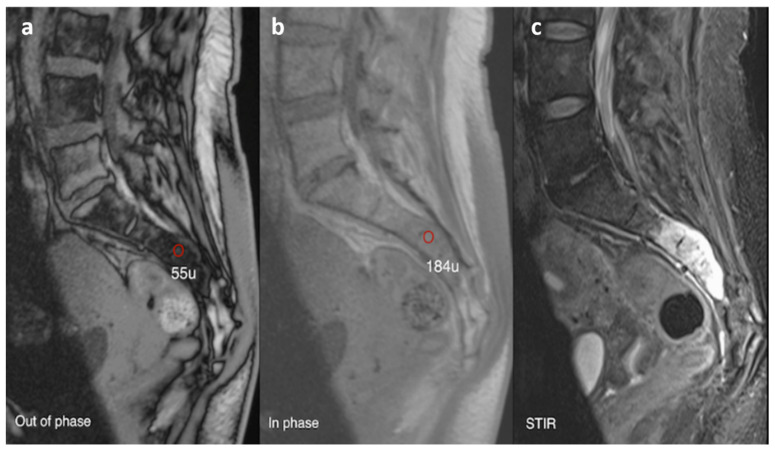
Sagittal out-of-phase (**a**), in-phase (**b**) and STIR (**c**) images of a sacral lesion. Regions of interest have been marked on the lesion. On the in-phase image the mean signal is 184 U, on the out-of-phase image the signal is 55 U. This shows that there is significant dropout (>20%) indicating a large amount of fat in the lesion and is therefore likely to be benign. The lesion turned out to be a sacral haemangioma.

Typically, haemangiomas are hyperintense on both T1 and T2/fluid-sensitive sequences in relation to the vertebral marrow, owing to their high fat content and slow vascular flow (Figure 14a,b). The areas of trabecular thickening appear as multiple lines and dots of signal voids on sagittal and axial imaging, respectively. Contrast is often not required for diagnosis, but if given, the lesions show enhancement [3]. Atypical haemangiomas, which can be highly vascular and contain little fat, may demonstrate some unusual signal patterns, with low to intermediate T1 signal. Chemical shift imaging with in-phase and opposed-phase sequences is also a useful adjunct in diagnosis and distinguishing from malignant lesions. While not a definitive feature, a signal intensity drop of >20% on the opposed phase imaging, suggesting presence of significant intralesional fat, favours a haemangioma (Figure 15a–c) [35].

**Figure 16:**
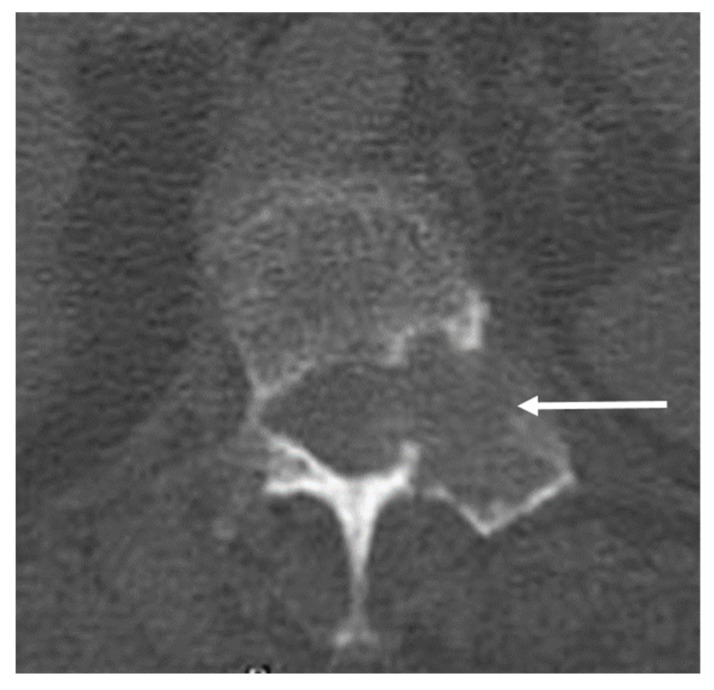
Axial CT showing an aggressive haemangioma of a thoracic vertebra (white arrow). Note the lytic appearance.

**Figure 17:**
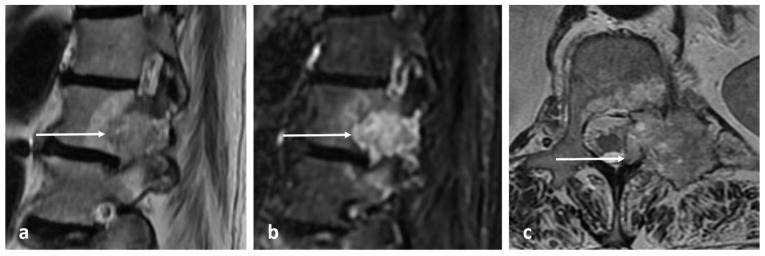
Sagittal T2 (**a**), STIR (**b**) and axial T2 (**c**) images showing an aggressive haemangioma of a thoracic vertebra (white arrows). The osseous destruction and extra osseous soft tissue component is well demonstrated on MRI.

**Figure 18:**
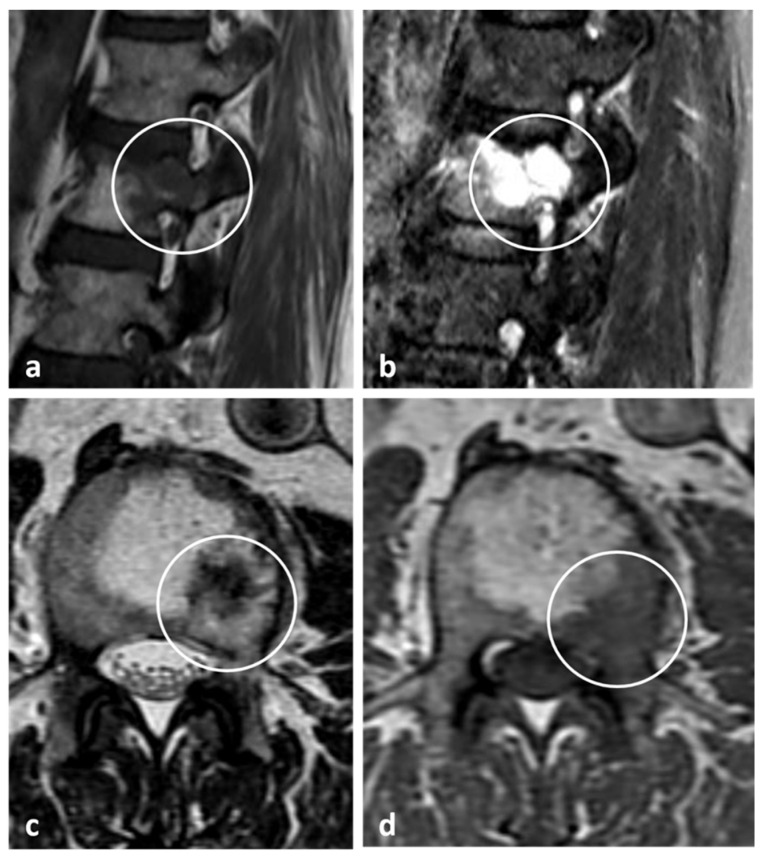
Sagittal T2 (**a**), STIR (**b**), axial T2 (**c**) and T1 (**d**) images showing a renal carcinoma metastasis within a haemangioma, known as a collision lesion (white circles).

On the contrary, aggressive haemangiomas may lack the typical fatty signal changes seen in conventional haemangiomas, and appear hypointense on T1 sequences, and may have an extraosseous soft tissue component (Figure 16 and Figure 17a–c). Enhancement may be seen. These may present a diagnostic challenge, as the lesions can be mistaken for a malignant entity. In very rare circumstances, secondary lesions can occur within a typical haemangioma, such as the presence of an atypical (low-fat) haemangioma within a typical haemangioma, or a metastatic deposit within a haemangioma, which can give rise to unusual signal patterns (Figure 18a–d). These are termed collision lesions and can present a diagnostic challenge. In these instances, biopsy may be required for definitive confirmation [36]. Vertebroplasty can be performed for painful haemangiomas.

While conventional haemangiomas usually do not require any management or follow-up, aggressive haemangiomas may require active management due to them being symptomatic and local mass effects. Management options include surgical resection, radiotherapy, embolization and percutaneous ablation [37].

### 3.8. Simple Bone Cyst

Simple bone cysts (SBC) are extremely rare in the spine, with only very few reported cases [38]. The commonly occur in the first and second decades of life [39]. The cervical and lumbar spine are most commonly affected, and they mainly involve the vertebral body or spinous process. Although the lesions themselves are asymptomatic, they can predispose to vertebral fracture and collapse, which can give rise to symptoms.

**Figure 19:**
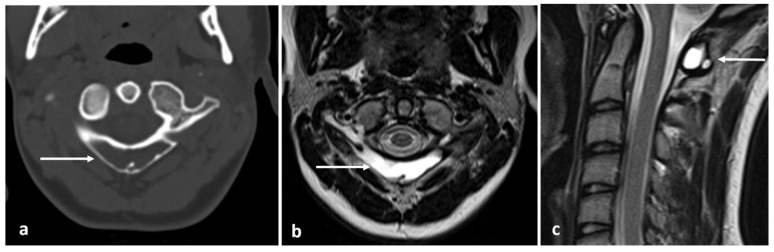
Axial CT (**a**), T2 (**b**) and sagittal T2 (**c**) images of a simple bone cyst of the C1 vertebra (white arrows). Note the lytic appearance on CT and fluid signal on T2 sequences.

In CT and MRI, they appear as well-defined cystic lesions within the vertebra. In MRI, the lesion follows normal fluid signal, hypointense on T1 and hyperintense on fluid-sensitive sequences [3] (Figure 19a–c).

Due to their rarity, a definitive consensus does not exist with regard to management approach. While the cysts themselves are indolent and can be conservatively managed, surgical curettage and bone grafting may be considered due to risk of fracture and to preserve spinal stability [38].

### 3.9. Aneurysmal Bone Cyst

Aneurysmal bone cysts (ABCs) are the third commonest benign bone tumour and frequently (up to 30%) occur in the vertebral column. There is a particular predilection for lumbar vertebrae and posterior elements [40]. The majority of primary ABCs occur in the first two decades; a secondary ABC should be considered in older patients [40]. Most ABCs have been shown to contain cytogenetic abnormalities in keeping with true neoplasms, and while rare cases of malignant transformation have been reported, these are rare and perhaps controversial [41].

**Figure 20:**
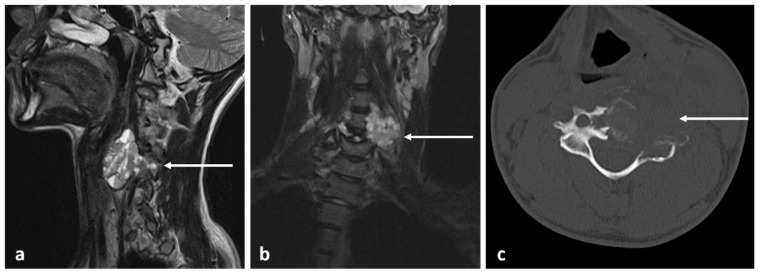
Sagittal T2 (**a**), coronal STIR (**b**) and axial CT (**c**) images of a cervical vertebral aneurysmal bone cyst (white arrows). The expansile lytic appearance on CT and multicystic appearance with fluid levels on MRI are typical features.

ABCs are expansile and thin the adjacent bone cortex, while also expanding it, often giving a “balloon”-like lucent appearance. ABCs can be locally aggressive. Focal cortical destruction with extension beyond the cortex is frequent [42]. In CT, ABCs demonstrate a narrow zone of transition (Figure 20c). The capsule and septa demonstrate contrast enhancement. Thin, bony septations may be present, but any osteoid formation must be assumed to be secondary due to telangiectatic osteosarcoma until proven otherwise [43].

ABCs are often identified on plain film, appearing as an expansile lucent lesion. The “absent pedicle” sign is a non-specific sign of a posterior element lesion, which can be present in posterior element ABCs. There can be a risk of pathological fracture.

MR appearances are defined by the classical fluid–fluid level appearances secondary to blood products in cysts of varying size (Figure 20a,b). The ABC should be surrounded by low signal in keeping with adjacent capsules. Peritumoral oedema may be present. Portions of the tumour may appear solid; the solid portions and septations enhance. Rarely, the ABC may appear entirely solid; solid ABCs are of intermediate T1 and T2 signal with a narrow zone of transition and no identifiable fluid–fluid levels nor septations. Of note, only 12 cases of solid spinal ABCs have been reported in the literature [44].

Similar to other benign vertebral lesions, symptoms are usually secondary to pressure on adjacent structures, including the spinal cord and vertebral arteries [45]. The usual treatment is surgical with excision or curettage [46]. Selective embolization has also shown to be effective in cases with a definite diagnosis and no pre-existing complications [47,48]. CT-guided sclerotherapy is also an effective and minimally invasive alternative to surgical treatment [49].

### 3.10. Notochordal Tumours

Benign notochordal tumours of the spine, also known as notochordal rests, represent residual foci of embryonic notochordal tissue. In the majority of cases, the rests are microscopic and thus occult on imaging. A small minority can be identified on imaging. The majority of these lesions are asymptomatic and incidentally identified [50]. A small proportion of patients may present with back pain and/or coccydynia. While the lesions arise from the same entity that gives rise to chordoma, these lesions are histologically distinct from each other. The lesions are fairly common and identified in up to 20% of autopsies according to one study [51]. Due to their embryonic origin, they usually occur in the vertebral bodies in the midline, usually adjacent to the endplate. Diffuse vertebral involvement may be present. The distribution is similar to that of chordomas, and hence is most likely seen in the sacrum.

**Figure 21:**
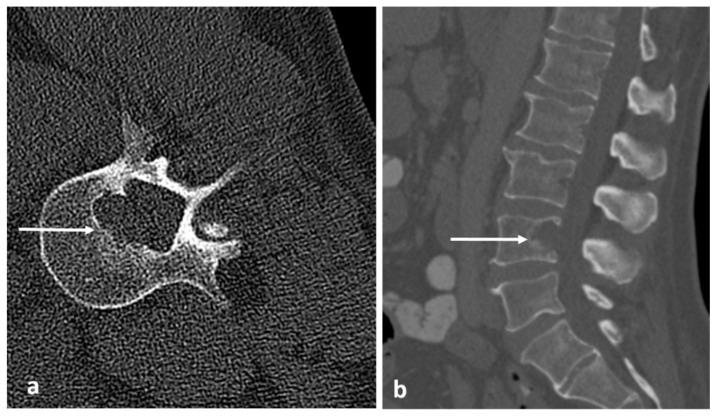
Axial (**a**) and sagittal (**b**) CTs of a benign notochordal remnant of L3 (white arrows). While most are non-destructive, this lesion was lytic.

**Figure 22:**
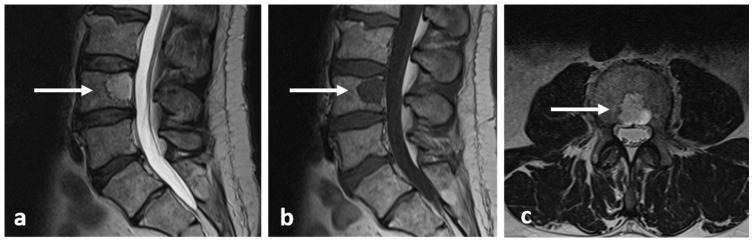
Sagittal T2 (**a**), T1 (**b**) and axial T2 (**c**) images of a benign notochordal remnant of L3 (white arrows). The central location within the vertebral body, and the low T1 and high T2 signal are typical features.

In CT, mild vertebral sclerosis or trabecular thickening can be seen, but occasionally can be lytic (Figure 21a,b). In MRI, they have low T1 and high T2 signal (Figure 22a–c). The lesions do not enhance with gadolinium contrast. Importantly, the bone configuration is usually maintained without expansion or destruction; however, some lesions may appear lytic with a narrow zone of transition. Unlike with chordoma, no soft tissue component is present. Biopsy may be required for definitive confirmation [51,52].

They do not usually warrant any intervention unless they cause significant pain, in which case surgical resection may be considered. Some authors have suggested periodic imaging follow-up in conservatively managed cases, due to a potential yet debatable risk of malignant transformation into chordoma [52].

### 3.11. Giant Cell Tumour (Benign Variant)

Giant cell tumours (GCTs) involve the spine in 7% of cases, and the vast majority (90%) involve the sacrum. They account for 9–15% of all primary spinal tumours. Peak incidence is during the third and fourth decades of life [3]. It is the most aggressive primary benign tumour of the spine, with a high recurrence rate. Their locally aggressive nature means patients commonly experience pain and neurological symptoms due to spinal cord and neural compression. A small subset of GCTs can be malignant; however, the benign forms which comprise the majority will be the focus of this discussion. Imaging features, however, can be very similar among the two types.

**Figure 23:**
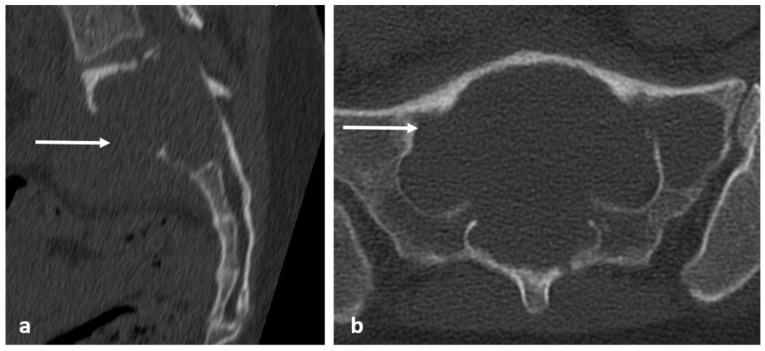
Sagittal (**a**) and axial (**b**) CT images of a sacral giant cell tumour (white arrows). The lesions typically appear lytic with thin peripheral shell of bone.

**Figure 24:**
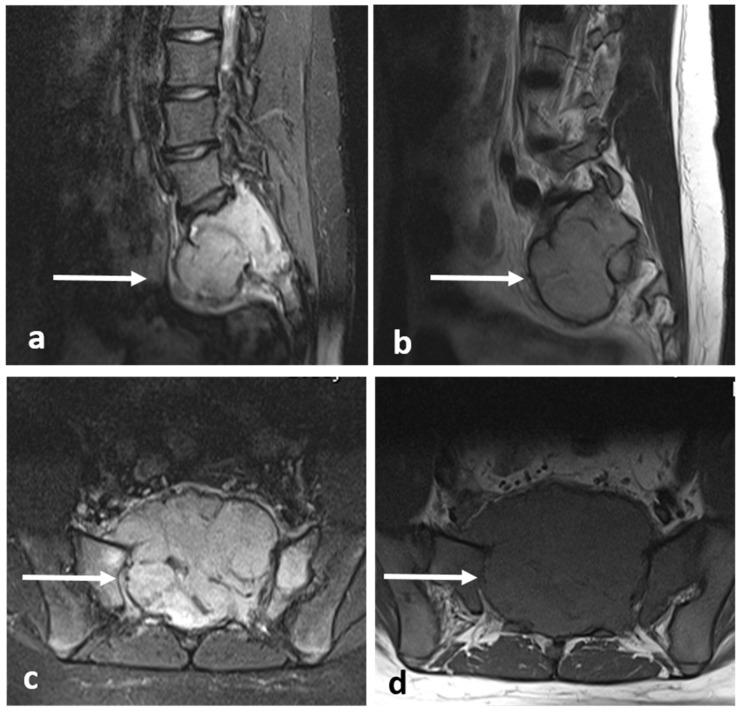
Sagittal STIR (**a**), T1 (**b**), axial STIR (**c**) and T1 (**d**) images of a sacral giant cell tumour (white arrows). There is intermediate to low signal intensity on both T1 and T2.

On imaging, they present as mixed cystic and solid expansile lesions, and usually involve the vertebral body. In CT, they appear lucent with thin peripheral shell of bone (Figure 23a,b); the cystic components tend to be multiloculated and this gives rise to a characteristic ‘soap bubble’-like appearance. In MRI, the tumour itself shows intermediate to low signal intensity on both T1 and T2 (Figure 24a–d), the latter usually being due to the presence of fibrosis and haemosiderin from repeated haemorrhage. Secondary aneurysmal bone cyst change is a common finding, and fluid–fluid levels can be appreciated via MRI. The soft tissue components are best appreciated in MRI and demonstrate enhancement. Surrounding marrow oedema is often seen on STIR. Vertebral collapse and extraosseous soft tissue extension are also common features [3,53].

Due to their high rate of recurrence, management can be challenging. A range of management options are currently employed, including curettage; surgical resection, including en-bloc resection; cryoablation and also medical treatment with Denosumab. Despite their benign nature, GCT can metastasise to lungs in 3–7% of cases, and screening with CT chest is prudent [54].

### 3.12. Eosinophilic Granuloma

Eosinophilic granuloma (EG) is a localised form of Langerhan’s cell histiocytosis (LCH) and typically affects children under the age of 10 years but is occasionally seen in young adults. About 15% of solitary EGs involve the spine. Patients typically present with pain, which may be exacerbated by pathological fracture [3].

**Figure 25:**
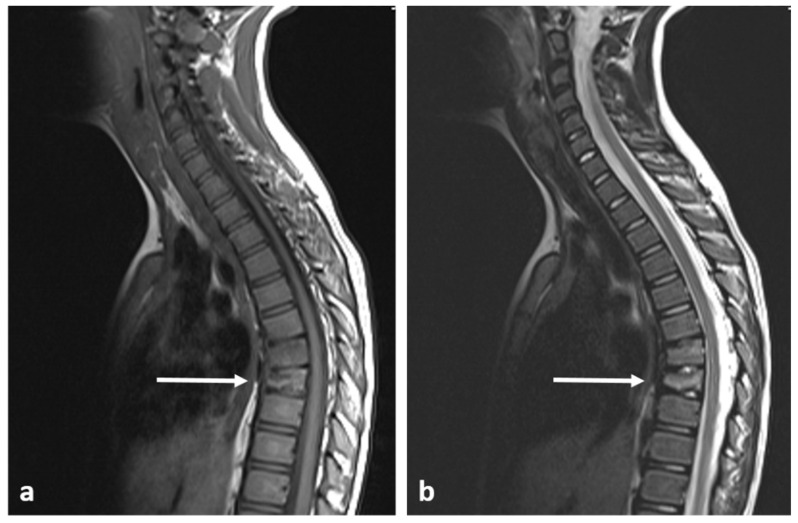
Sagittal T1 (**a**) and T2 (**b**) images of an eosinophilic granuloma of a mid-thoracic vertebra (white arrows). The signal characteristics are usually non-specific, with lesions appearing hypo to isointense on T1 and hyperintense on T2. Pathological collapse, as seen here, is common.

EGs usually involve the vertebral body with preservation of the posterior elements. Thoracic spine is the commonest site of involvement, followed by the lumbar and cervical spine. In half the cases, only a single vertebra is affected. Pathological collapse is common. Vertebra plana is a characteristic feature, although this is only seen in about 40% of cases. Radiographs and CT demonstrate a lucent lytic lesion with bone destruction. An extra-osseous soft tissue component may be seen. MRI signal characteristics are non-specific, with lesions typically being hypo- to isointense on T1 and hyperintense on T2 and STIR sequences (Figure 25a,b). There is often diffuse enhancement in post contrast imaging [3,55].

EGs have an excellent prognosis, particularly when solitary. The majority of lesions resolve via fibrosis in 1–2 years. However, surgical excision may be necessary when there are local compressive effects or when symptoms persist. Radiotherapy, chemotherapy and percutaneous ablation are also used [56].

## 4. Conclusions

Imaging plays a critical role in the detection and characterization of primary benign neoplasm of the spine. CT and MRI are the main modalities used, and understanding the characteristic imaging features of these lesions is important in the interpretation of these images (Table 2).

## Figures and Tables

**Table 1:** Classification and types of primary benign vertebral tumours.

Classification	Tumour
Osteogenic tumours	Osteoma (enostosis)
	Osteoid osteoma
	Osteoblastoma
Chondrogenic tumours	Osteochondroma
	Chondroblastoma
Vascular tumours	Haemangioma, including aggressive haemangioma
Osteoclastic giant cell-rich tumours	Aneurysmal bone cyst
	Benign Giant cell tumour
Notochordal tumours	Notochordal rest
Other mesenchymal tumours of bone	Simple bone cyst
	Fibrous dysplasia
Haematopoetic neoplasms	Eosinophilic granuloma

**Table 2:** A summary of the pertinent and typical CT and MRI features of the aforementioned primary benign vertebral tumours.

Type of Tumour	CT Features	MRI Features
Osteoma	Dense sclerotic lesion. Hounsfield density >885 a helpful indicator but not definitive.	T1 and T2, STIR hypointense due to sclerosis.
Osteoid osteoma	Lucent nidus, usually 2–10 mm. May be surrounded by sclerotic rim.	Surrounding marrow oedema on fluid sensitive sequences (best seen on STIR). Nidus can be occult on MRI.
Osteoblastoma	Expansile lucent lesion. Sclerotic rim. Bone destruction. Variable intralesional ossification.	Low to intermediate T1 signal and intermediate to high T2 signal. Surrounding soft tissue oedema, extra osseous soft tissue component.
Fibrous dysplasia	Ground glass matrix. Cystic areas can appear lytic.	Low on T1 and intermediate to high on T2. Cystic areas are hyperintense on T2 and STIR.
Osteochondroma	Lesion continuity with cortex and medulla.	Cartilage cap
Chondroblastoma	Osteolytic lesion. Variable intralesional calcification (chondroid matrix)	Extraosseous soft tissue component
Haemangioma	Honey comb appearance. ‘Corduroy’ and ‘polka dot’ signs.	Usually T1 and T2 hyperintense due to fat content. Signal drop out >20% on out of phase chemical shift imaging.
Simple bone cyst	Well defined lucent rim with narrow zone of transition.	Fluid signal lesion. May have some internal haemorrhage (high T1 signal).
Aneurysmal bone cyst	Expansile lytic lesion with internal bone septations.	Fluid—fluid levels within the cysts, high T1 signal within layering fluid content due to haemorrhage.
Benign notochordal tumour	Midline lesion in the body. Sclerosis or trabecular thickening typically, but lysis can be present.	Low T1 and high T2 signal. No enhancement.
Giant cell tumour	Mixed cystic and solid expansile lesions, with thin peripheral bony shell. ‘Soap bubble’ appearance.	Secondary ABC change is common, with fluid—fluid levels. Intermediate to low signal intensity on both T1 and T2. Enhancing soft tissue component.
Eosinophilic granuloma	Lucent lytic lesion, with bone destruction. Vertebra plana.	Non-specific appearance, with low T1 and high T2 signal. Diffuse enhancement.

## Data Availability

Not applicable.
